# Progress in Medicine

**Published:** 1897-09-25

**Authors:** 


### Progress in Medicine.

### DISEASES OF THE BLOOD.

**Pernicious Anæmia.**—Recent work on this disease, as detailed in successive articles in The Hospital, has given a fairly complete picture of the symptoms, while the net results have left both the true pathology and treatment unsettled. Thus[1] we know that it specially attacks middle-aged persons, males, perhaps more often than females. It is often fatal, and accompanied by hæmorrhages; the red cells are greatly reduced in number, while the hæmoglobin is less affected, but many of the red cells are abnormally large, and others irregular in shape, while some are nucleated. The proteids and specific gravity of the serum, according to Adami, are diminished, while the globulins are increased relatively to the albumen. Finally the red marrow becomes more like that found in children. Bone marrow, arsenic in increasing doses, and oxygen inhalations have at times seemed to be successful, and in other cases have failed entirely. Repeated transfusion of small quantities of blood, or the daily injection of five milligrammes of corrosive sublimate, have been followed by cures in a few instances, while intestinal antisepsis seems to be a complete failure. The disease may show considerable pyrexia, and be at first difficult to distinguish from typhoid or tuberculosis. Some help for a diagnosis from these is gained, both by the absence of physical signs and rashes, and by the duration of the illness. Still more important are the retinal hæmorrhages,[2] which are so common in pernicious anæmia. The normal size of the spleen, again, excludes splenic anæmia, and the same sign with the absence of any increase in white corpuscles negatives leucocythæmia. Neither do we see the groups of enlarged glands (with but slight loss of red cells compared to that of hæmoglobin) which are found in Hodgkin's disease. The diagnosis may have to be made also from cancer, Bright's disease, secondary hæmorrhage, and Addison's disease, with little or no pigmentation, but usually the symptoms given above are sufficient to establish a safe decision. Diarrhœa, vomiting, and dyspnœa are frequent concomitants, and tax our resources in combating them.

Among recent studies of this disease we may refer to Dieballa's[3] account of a case where variation in the number of leucocytes in the blood had taken place, being fewer from diminution of the polynuclear cells. Usually the eosinophiles are much reduced, but in one instance he has found them present to the amount of 8 per cent. Here, however, where the reduction of white cells was only moderate and the eosinophiles at least normal, he considers that there could have been only a moderate change in the spleen and bone marrow. Gumprecht[4] found that rapid degeneration takes place in the leucocytes both in this disease and in leucæmia. From 1 to 10 per cent. are found thus altered in various ways, chiefly with respect to the nucleus. Disintegration of leucocytes is almost entirely absent in normal blood except in the polynuclear elements. This destruction and breaking up of the nucleus has been postulated by the chemists for a long while, at least in leucocythæmia, from the fact that there is an increased elimination of uric acid and alloxur bodies. Byron Bramwell[5] reports a rapidly progressive case of pernicious anæmia associated with marked jaundice, which made an equally rapid recovery under large doses of arsenic. The patient had not been really ill for more than six weeks before his admission, when the red corpuscles numbered only 810,000. Bramwell determined to test the question of the existence of any organisms in the blood in this case, as the symptoms appeared so acute, but none were found. Still, he remarks there may be different varieties of the disease, and his results by no means prove the universal absence of such microbes in all cases. He notices, too, the great numbers of Eichhorst's corpuscles in the blood, which are small deeply-pigmented bodies; the intensity of the jaundice, though the stools were only of a deep orange or ochre colour, and there was merely a trace of pigment in the urine, which was never very dark; the copious deposit of uric acid in the urine, and the temporary presence of albumen and a few casts; finally, the absence of all parasitic organisms in the stools, and the large doses of arsenic, amounting to 50 and 60 drops daily, which the man took, and under which rapid disappearance of all symptoms was observed. After six weeks' treatment he left the hospital in good health with 3,320,000 corpuscles, which were still more increased before the date of writing.

**Hodgkin's Disease.**—Butlin[6] argues that though in some cases the histological structure of the glands does not differ from that of ordinary enlarged glands, yet in others we have a totally different form, so that possibly there are here also two distinct diseases confounded under one name. In some patients we see the affection running an exceedingly slow course, and the tumours varying in size from time to time. Thus he refers to a case which he watched for ten years. The effect of arsenic, too, is remarkable in many of the patients, the glands disappearing under large continued doses as if by magic, while in others it has little or no effect. In these points there is a great difference from the behaviour of malignant growths, at any rate in one type of the disease. Frederick Eve[7] discusses four cases of a special type. He draws attention to the length of time during which the disease lasted, which in one patient was ten years; to the mobility of the glands for long periods, and the easiness with which they could be shelled out, as opposed to the firm adhesions of tuberculous glands to each other and to the surrounding structures. Indeed, the disease may remain localised for a long while in one region without affecting distant glands or the general system, and herein differs from the acute form. Still, he, unlike Butlin, finds no microscopical difference in the gland structures of either group, nor between the hard and soft varieties. In the acute forms we more often see, indeed, large polynuclear cells, and some growths may be entirely composed of young cells, while others have mature lymph cells with a ring of protoplasm around the nucleus mingled with them. Finally he refers to Delbet's discovery of bacilli in the spleen of a case of lymphadenoma, which were cultivated and injected into a dog. In this animal the disease was reproduced, and the bacillus demonstrated in its blood. The whole question of the varieties of lymphadenoma and their relations to malignant new growths on the one hand, and to infective processes on the other, is by no means clear, though a subject of the greatest interest.

[1] B. Med. Jour., May 15. [2] Clin. Jour., June 23. [3] Amer. Jour. Med. Sc., April. [4] Med. Rec., Jan. 16. [5] Lancet, July 24. [6] Clinic. Jour., June 23. [7] B. Med. Jour., March 6.

